# Predicting the therapeutic role and potential mechanisms of Indole-3-acetic acid in diminished ovarian reserve based on network pharmacology and molecular docking

**DOI:** 10.1186/s41065-024-00348-6

**Published:** 2024-11-21

**Authors:** Jianxiu Zheng, Liyan Wang, Ahui Liu, Haofei Shen, Bin Wang, Yanbiao Jiang, Panpan Jing, Defeng Guan, Liulin Yu, Xuehong Zhang

**Affiliations:** 1 Lanzhou University, Chengguan District, No. 222 Tian Shui South Road, Lanzhou, Gansu, 730000 People’s Republic of China; 2 The First School of Clinical Medicine, Lanzhou University, Chengguan District, No. 1, Dong Gang Xi Road, Lanzhou, Gansu, 730000 People’s Republic of China; 3 The First Hospital of Lanzhou University, Chengguan District, No. 1 Dong Gang Xi Road, Lanzhou, Gansu, 730000 People’s Republic of China; 4 Key Laboratory for Reproductive Medicine and Embryo, Gansu Province, Lanzhou, People’s Republic of China

**Keywords:** Indole-3-acetic acid (IAA), Diminished ovarian reserve, Network pharmacology, Molecular docking, Molecular dynamics simulation

## Abstract

**Background:**

Indole-3-acetic acid (IAA), an indole analog produced by intestinal microorganisms metabolizing tryptophan, has anti-inflammatory and antioxidant properties and thus has potential applications in ovarian protection, although the exact mechanism is unknown. The present study preliminarily investigated the pharmacological mechanism of IAA in alleviating diminished ovarian reserve (DOR) by network pharmacology and molecular docking.

**Methods:**

Relevant target proteins of IAA were searched in SwissTargetPrediction, PharmMapper, TargetNet, BATMAN-TCM, and SuperPred databases. The potential targets of DOR were obtained from GeneCards, DisGenet, OMIM, and Drugbank databases. Both common targets were then imported into the String website to construct a PPI network, and these targets were analyzed for GO and KEGG enrichment. Finally, we utilized molecular docking to validate the possible binding conformations between IAA and the candidate targets. We used in vitro experiments to preliminarily investigate the effects of IAA on DOR.

**Results:**

We obtained 88 potential targets for IAA and DOR interaction. We received 16 pivotal targets by constructed protein interaction screening. KEGG enrichment analysis mainly included the AGE-RAGE signaling pathway, IL-17 signaling pathway, Chemical carcinogenesis—reactive oxygen species in diabetic complications, etc. GO functional analysis showed that IAA treatment of DOR may involve biological processes such as response to external stimuli, hypoxia, gene expression, and regulation of enzyme activity. Molecular docking and in vitro experiments further revealed the potential effects of IAA on MMP2, TNF-α, AKT1, HSP90AA1, and NF-κ B.

**Conclusion:**

We preliminarily revealed the potential protective effects of IAA against DOR through multiple targets and pathways, which provides a new research strategy for the molecular mechanism of IAA to alleviate DOR in the future. However, further studies need to demonstrate whether IAA can be used as a compound to prevent and treat DOR.

**Supplementary Information:**

The online version contains supplementary material available at 10.1186/s41065-024-00348-6.

## Introduction

Diminished ovarian reserve (DOR) is defined as a decrease in the number and quality of oocytes [1]. DOR is a significant challenge in the current fertility field, mainly due to its poor outcome in assisted reproduction, which is primarily characterized by low ovarian responsiveness to ovarian stimulation in vitro fertilization-embryo transfer, low egg acquisition rate, low fertilization rate and an increased risk of early miscarriage and embryo termination [2–4]. The pathogenesis of DOR is currently unknown, and it may be related to inherited genetic mutations, DNA methylation levels, mitochondrial dysfunction, infection, and medical or environmental factors [5, 6]. The age of patients with DOR is gradually getting younger. DOR has become a popular research area in the field of reproductive health, but there is no optimal treatment option for patients with DOR [7].

Granulosa cells can provide oocyte nutrients and signaling molecules through gap linkage and transzonal projection (TZP) and secrete paracrine signals to influence oocyte maturation and meiosis [8, 9]. ROS positively affects oocyte development and ovulation, but oxidative stress occurs when there is an imbalance between ROS and antioxidant systems. Oxidative stress is one of the causes of ovarian dysfunction, which can lead to follicular atresia and abnormal meiosis of oocytes, shortened chromosomal telomeres, reduced embryonic developmental potential, and, ultimately, low fertility [10]. Maintaining the balance of the body’s oxidative and antioxidant systems, supplementation with antioxidants may attenuate a series of injuries caused by excessive accumulation of ROS and positively affect the quality of oocytes and ovarian reserve function.

Indole-3-acetic acid (IAA) is a plant growth regulator; however, it has been demonstrated that mammalian intestinal microorganisms metabolize tryptophan to produce IAA, which can be absorbed into the bloodstream to reach various organs and tissues. IAA has anti-inflammatory and antioxidant effects in tissues and cells associated with nonalcoholic fatty liver disease [11], ankylosing spondylitis [12], and antioxidant dental pulp stem cells [13]. At the same time, our previous study found significantly lower levels of tryptophan and its indole metabolites IAA and IPA in the follicular fluid of infertile women with decreased ovarian reserve function compared to infertile women with normal ovarian reserve function [14]. However, the relevant role and biological mechanisms regarding IAA in ovarian reserve hypoplasia have not been studied.

**Fig. 1:**
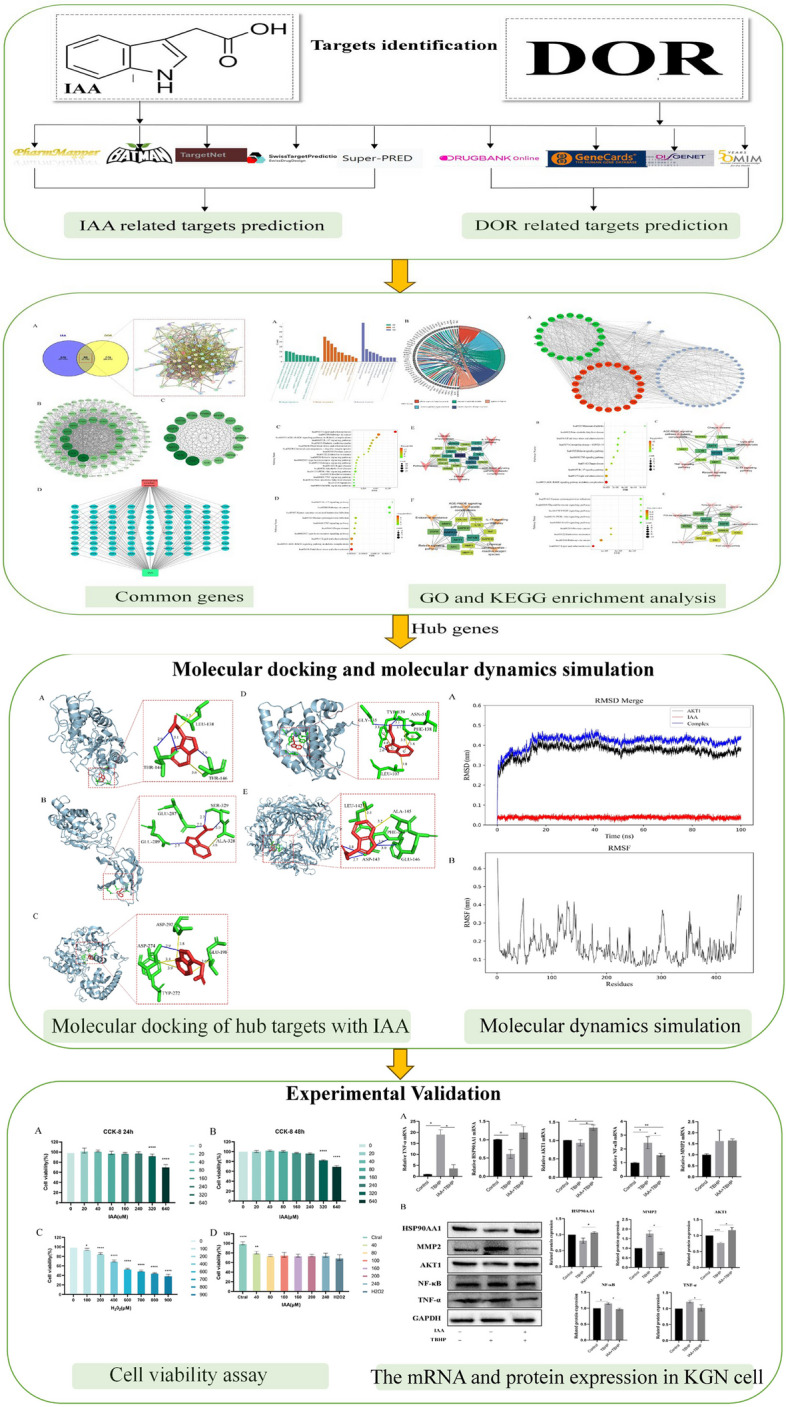
Workflow chart

Network Pharmacology is a multidisciplinary research field based on systems biology, genomics, proteomics, and other disciplines, which is a method to explore new drug targets and molecular mechanisms by combining computer analysis simulation with in vivo and in vitro experiments and integrating a large amount of information [15]. This research explores the potential targets and molecular mechanisms of IAA to improve ovarian reserve based on network pharmacology. The experimental flow is shown in Fig. 1.

## Materials and methods

### IAA target prediction

The SMILES numbers and 2D structures of IAA were obtained in PubChem (https://pubchem.ncbi.nlm.nih.gov/) [16]. In SwissTargetPrediction (http://swisstargetprediction.ch/) [17], PharmMapper (http://www.lilab-ecust.cn/pharmmapper) [18], TargetNet (http://targetnet.scbdd.com) [19], BATMAN-TCM (http://bionet.ncpsb.org.cn/batman-tcm/index.php) [20], SuperPred (http://prediction.charite.de) [21]with “Homo sapiens” as the query condition to obtain IAA gene targets, and then UniProt (https://www.uniprot.org) [22]was used to standardize the IAA gene target names.

### DOR target acquisition

Using diminished ovarian reserve as the query condition, we searched relevant target genes in GeneCards (https://www.genecards.org/) [23], DisGenet (https://www.disgenet.org/) [24], OMIM (https://www.omim.org) [25], and Drugbank (https://go.drugbank.com) [26], selected target genes with high degree of correlation with the disease, and then standardized the IAA gene target names through UniProt.

### Mapping Venn diagrams and PPI protein interaction networks

Use Venny 2.1.0 (https://bioinfogp.cnb.csic.es/tools/venny) to map the intersecting targets of IAA and DOR and obtain the intersecting gene targets. The above intersecting gene targets were imported into STRING (https://cn.string-db.org) [27]network, Homo sapiens was selected as the species, the minimum required interaction threshold was set to medium confidence (medium confidence 0.4), and the isolated targets with no connection were removed. The intersecting gene targets with high correlation were imported into Cytoscape 3.10.1 to visualize the protein interactions network diagram. The intersecting gene targets greater than or equal to the median of degree, closeness centrality, and betweenness centrality were selected.

### GO and KEGG signaling pathway enrichment analysis

The key intersecting targets were imported into the DAVID(https://david.ncifcrf.gov) [28]data platform to obtain gene ontology (GO) functional analysis and Kyoto Encyclopedia of Genes and Genomes (KEGG) signaling pathway enrichment analysis, and then the results were imported into the bioinformatics.com.cn(http://www.bioinformatics.com.cn) for data visualization.

### Functional module analysis

The intersecting genes were subjected to functional module analysis using the MCODE plugin in Cytoscape 3.10.1 software, and the highest weighted nodes were taken as significant nodes based on the criteria set by the module. Subsequently, GO and KEGG enrichment analyses were done on the target proteins obtained from module aggregation.

### Molecular docking analysis

The sdf file of the 2D structure of IAA (Compound CID: 802) was obtained from the PubChem (https://pubchem.ncbi.nlm.nih.gov) database, and the OpenBabel-3.1.1 software was used to convert the sdf format to mol2 format. The structures of the core target proteins in the PPI network diagram were obtained in the RCSB PDB(https://www.rcsb.org) database and dewatered and removed from unnecessary ligands using PyMOL (version 2.5) software. Processing, such as hydrogenation, stance parameter adjustment, etc., were performed and converted to PDBQT format using Autodock Tools-1.5.7, Gasteiger charges were calculated, and binding pockets were identified. Molecular docking calculations were performed using AutoDock Vina, and the docking results were visualized using PyMOL and PLIP [29]. The stability of ligand-receptor binding was determined based on the Affinity (kcal/mol) value generated by docking.

### Molecular dynamics simulation

After molecular docking, we chose AKT1, which has the strongest binding activity to IAA, for molecular dynamics simulations. Molecular dynamics simulations were performed using the GROMACS package (version 2022.3), with the Amber99sb-ildn force field in Gromacs for target proteins and the GAFF force field for small molecules to obtain the appropriate molecular parameters. The simulation conditions were carried out at a static temperature of 300 K and atmospheric pressure (1 Bar). Amber99sb-ildn was used as a force field, water molecules were used as a solvent (Tip3p water model), and the total charge of the simulation system was neutralized by adding an appropriate number of Na + ions. The steepest descent method is first used to minimize the energy, and thereafter, 100,000 steps of isothermal isovolumetric tethered (NVT) and isothermal isobaric tethered (NPT) equilibrium are carried out with a coupling constant of 0.1 ps and a duration of 100 ps, respectively. Finally, the free molecular dynamics simulation was performed. The process consisted of 5,000,000 steps, the step length was 2 fs, and the total duration was 100 ns. We calculated the root mean square deviation (RMSD) and root mean square fuctuation (RMSF) values to assess the stability and fexibility of the complexes. The binding free energy values and interactions of ligands with proteins were calculated by the MM/GBSA method [30].

### Cell line culture

KGN cells were cultured in Procell’s KGN cell-specific medium (DMED/F12) (Procell, China), which contains 10% fetal bovine serum and 1% penicillin/streptomycin in a humidified incubator with 5% CO2.KGN cells were used from passages 3–20.

### Cell proliferation assays

According to the instructions, survival cell viability was assessed using the Cell Counting Kit-8 (CCK8; AbMole, USA) kit. After drug treatment, 10 μl of CCK8 solution was added to each well of the culture plate and placed in an incubator to culture the cells for 2 h, followed by absorbance detection at 450 nm. This absorbance is proportional to cell viability and allows quantitative determination of cell viability.

### Western blotting

Cells were lysed in RIPA tissue lysate (Biosharp, China) containing PMSF, phosphorylated protein inhibitor (A/B) (Servicebio, China) on ice for 15 min, followed by centrifugation at 12,000 rpm for 10 min. Protein samples were electrophoresed on 8% or 10% polyacrylamide gels and then transferred onto 0.22um PVDF (Immobilon Transfer Membrane) membranes, which were closed for 2 h at room temperature with 5% milk configured in Tween 20 (TBST) with GAPDH (proteintech, 60,004–1-IG), HSP90AA1 (wanleibio, WL01763), AKT-1 (proteintech, 10,176–2-AP), MMP2 (proteintech,10,373–2-AP), TNF-alpha (wanleibio, WL01581), and NF-κB (wanleibio, WL01917) were incubated overnight at 4 °C. After being washed three times with TBST, the cells were incubated with HRP-conjugated secondary antibody at room temperature for 2 h and then immersed in an ECL luminescent solution (Xin Saimei, China) and exposed to a BIO-RAD instrument (Bio-Rad, USA). Finally, the bands were analyzed for gray value using Image J.

### Real-time quantitative PCR (RT-qPCR)

Total RNA from KGN cells was extracted using M5 HiPer Total RNA Extraction Reagent (TRIgent) (Mei5bio, China) reagent. Extracted RNA was reverse transcribed using FastKing One-Step De-genomic cDNA First Strand Synthesis Premix Reagent (KR118) (TIANGEN, China). Real-time quantitative PCR (RT-qPCR) was performed using SuperReal Fluorescence Quantitative Premix Reagent Enhanced Kit (SYBR Green) (FP205) (TIANGEN, China) and AB Applied Biosystems instrument (ABI, USA). The primer sequences are shown in Table 1.

**Table 1:** Primer sequences

Gene name	Forward	Reverse
GAPDH	GGAAGCTTGTCATCAATGGAAATC	TGATGACCCTTTTGGCTCCC
TNF-α	GCTGCACTTTGGAGTGATCG	ATGAGGTACAGGCCCTCTGA
AKT1	ATGAGGTACAGGCCCTCTGA	CCCGGTACACCACGTTCTTCT
HSP90AA1	GAAGGAATTTGAGGGGAAGACTTTA	TGCCATGTAACCCATTGTTGAG
NF-κB	TGTAACTGCTGGACCCAAGGAC	CAAATAGGCAAGGTCAGGGTG
MMP2	AGTGGATGATGCCTTTGCTCG	CAAGGTCCATAGCTCATCGTCAT

### Statistical analysis

Data are represented as means ± SD with the student’s t-test used to calculate statistical significance between groups. *P* < 0.05 were considered significant.

## Results

### Retrieval of target genes for indole-3-acetic acid and diminished ovarian reserve

After removing duplicates, 414 indole-3-acetic acid target genes were retrieved by searching SwissTargetPrediction, PharmMapper, TargetNet, BATMAN-TCM, and SuperPred databases (Supplementary file 1). After eliminating duplicates, 864 diminished ovarian reserve gene targets were retrieved by searching GeneCards, DisGenet, OMIM, and Drugbank databases (Supplementary file 2).

### Obtaining intersecting gene targets of drugs and diseases and constructing PPI

**Fig. 2:**
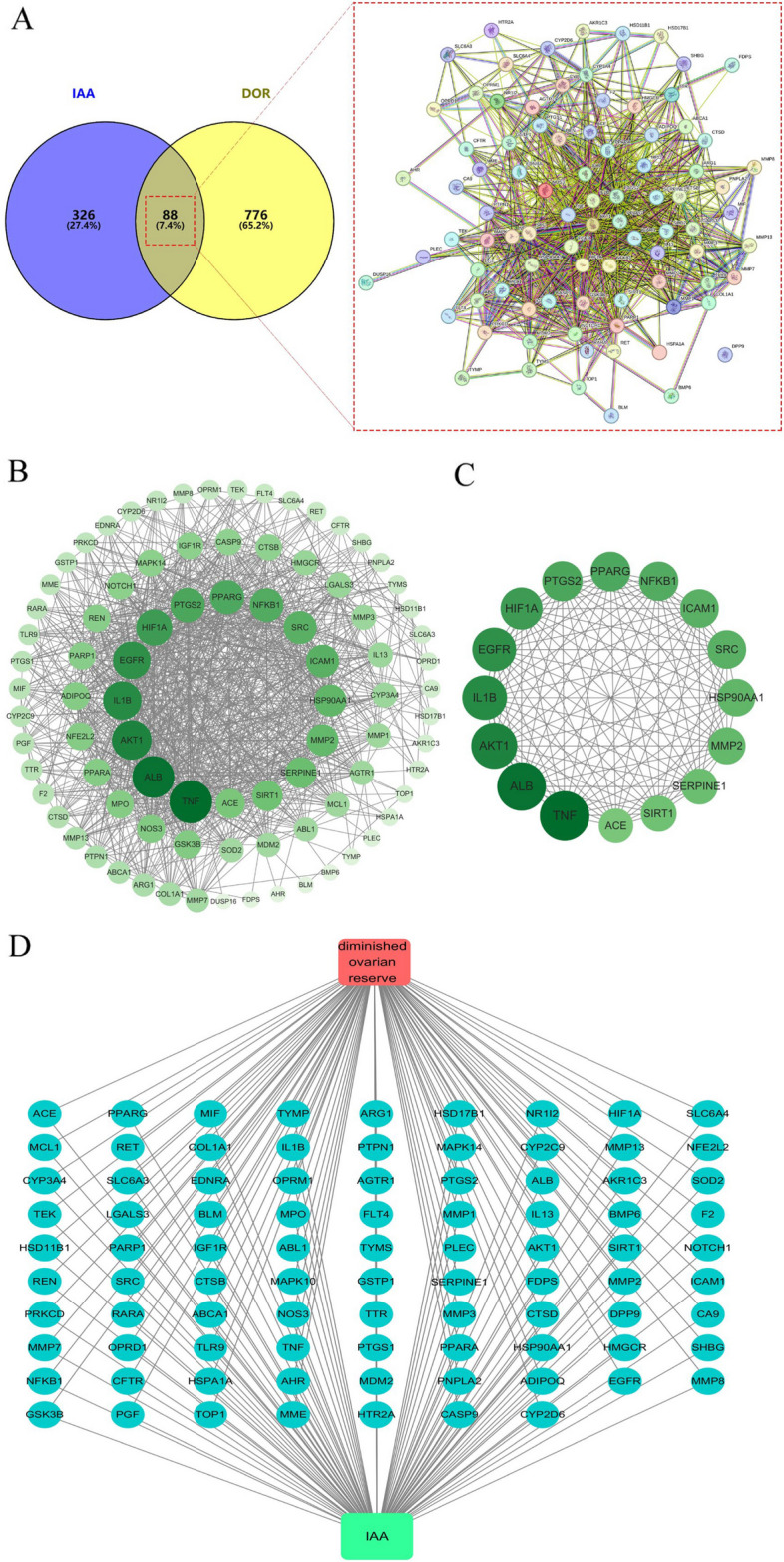
Venn diagram and PPI network of potential targets. **A** Venn diagram showing the common genes between IAA and DOR. **B** PPI network of potential targets. **C** 13 hub genes of IAA against DOR were identified by network topological parameters analysis (Degree, Closeness, and Betweenness). **D** IAA-target genes-DOR network

88 intersecting gene targets were obtained by Venny 2.1.0 (Fig. 2A). These 88 intersecting gene targets were imported into the String database. The confidence value was set to 0.4, and the remote targets were excluded from obtaining the PPI network graph (Fig. 2B). Subsequently, the topological parameters of each node protein were analyzed, and 16 central genes were obtained by screening based on the average values of Degree, Betweenness, and Closeness (35, 72.06897, and 0.605249, respectively) (Supplementary file 3). They included TNF, ALB, AKT1, IL1B, EGFR, HIF1A, PTGS2, PPARG, NFKB1, ICAM1, SRC, HSP90AA1, MMP2, SERPINE1, SIRT1, ACE (Fig. 2C). In addition to this, to more intuitively understand the pharmacological effects of IAA against DOR, disease-intersecting gene target-drug maps were drawn by Cytoscape 3.10.1 (Fig. 2D). Green squares represent drugs, red squares represent diseases, and blue circles represent intersecting target proteins.

### GO enrichment analysis

**Fig. 3:**
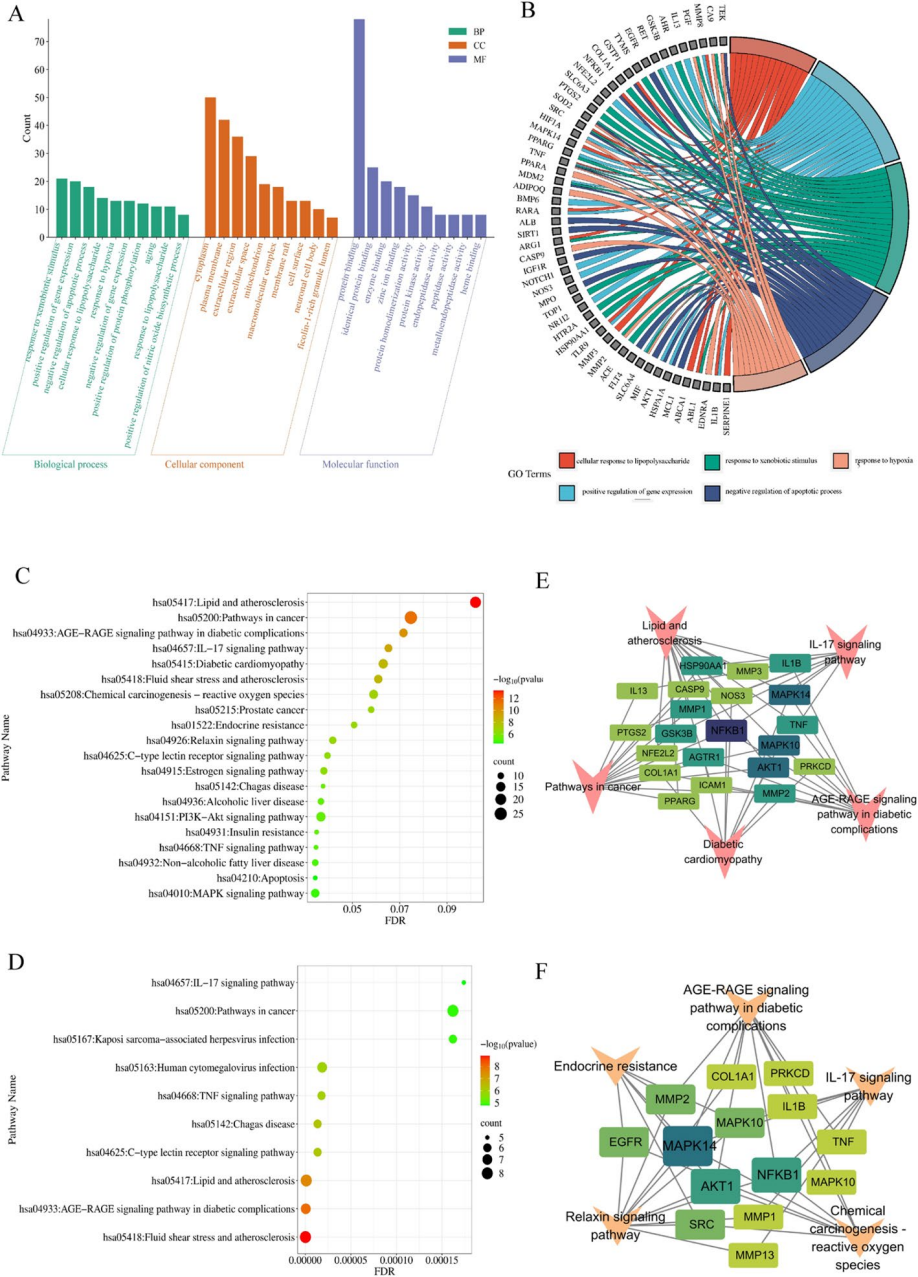
The GO and KEGG enrichment analysis for common targets. **A** GO enrichment analysis (The top 10 enriched terms of each part). **B** The top 5 of the biological processes. **C** KEGG enrichment analysis of therapeutic targets (the top 20 enriched pathways). **D **KEGG enrichment analysis of 16 core gene targets (the top 10 enriched pathways). **E**–**F** Target-KEGG pathway network. The orange V-shaped nodes represent the pathways, and the green square-shaped nodes represent the targets

The 88 intersected genes were imported into the DAVAD database for GO enrichment analysis. A total of 364 biological processes (BP), 52 cell components (CC), and 76 molecular functions (MF) were obtained with *P* < 0.05 as the screening condition. The results of screening the top 10 of the three entries according to the P value and the number of enriched genes are shown in Fig. 3A. Subsequently, GO chord plots were plotted for the first five enriched BPs (Fig. 3B). The BP results showed that the effects of IAA on DOR were correlated with the mechanisms of response to xenobiotic stimulus, positive regulation of gene expression, cellular response to lipopolysaccharide, response to hypoxia, negative regulation of apoptotic process, and mechanism of aging. In addition, 10 of the 16 core hub genes were also enriched in the first 5 BPs, including EGFR, NF-κB, PTGS2, SRC, HIF1A, PPARG, TNF-α, ALB, MMP2, and AKT1. The molecular functions are mainly related to a series of biological functions such as enzyme binding, endopeptidase activity, zinc ion binding, peptidase activity, and protein kinase activity. Meanwhile, cellular components such as the extracellular region, macromolecular complex, cytoplasm, and plasma membrane were also annotated using GO analysis.

### KEGG enrichment analysis

KEGG enrichment analysis of 88 therapeutic targets yielded 123 pathways (*P* < 0.05). After data screening, we identified the top 20 pathways of IAA for DOR (Fig. 3C), which were mainly enriched in the AGE-RAGE signaling pathway in diabetic complications、IL-17 signaling pathway、Chemical carcinogenesis—reactive oxygen species、Endocrine resistance、Relaxin signaling pathway、Estrogen signaling pathway 、PI3K-Akt signaling pathway、Insulin resistance、TNF signaling pathway 、MAPK signaling pathway、Apoptosis. In addition, to further understand the possible signaling pathways by which IAA affects DOR, we did a KEGG analysis of the 16 core gene targets, whose top 10 enriched pathways also included the AGE-RAGE signaling pathway in diabetic complications, the TNF signaling pathway, the IL- 17 signaling pathway (Fig. 3D). Meanwhile, to visualize the critical targets of action in the first five enrichment pathways involved in the regulation of DOR by IAA, we therefore constructed the Target-KEGG pathway network (Fig. 3E-F).

### Functional module-based network analysis

**Fig. 4:**
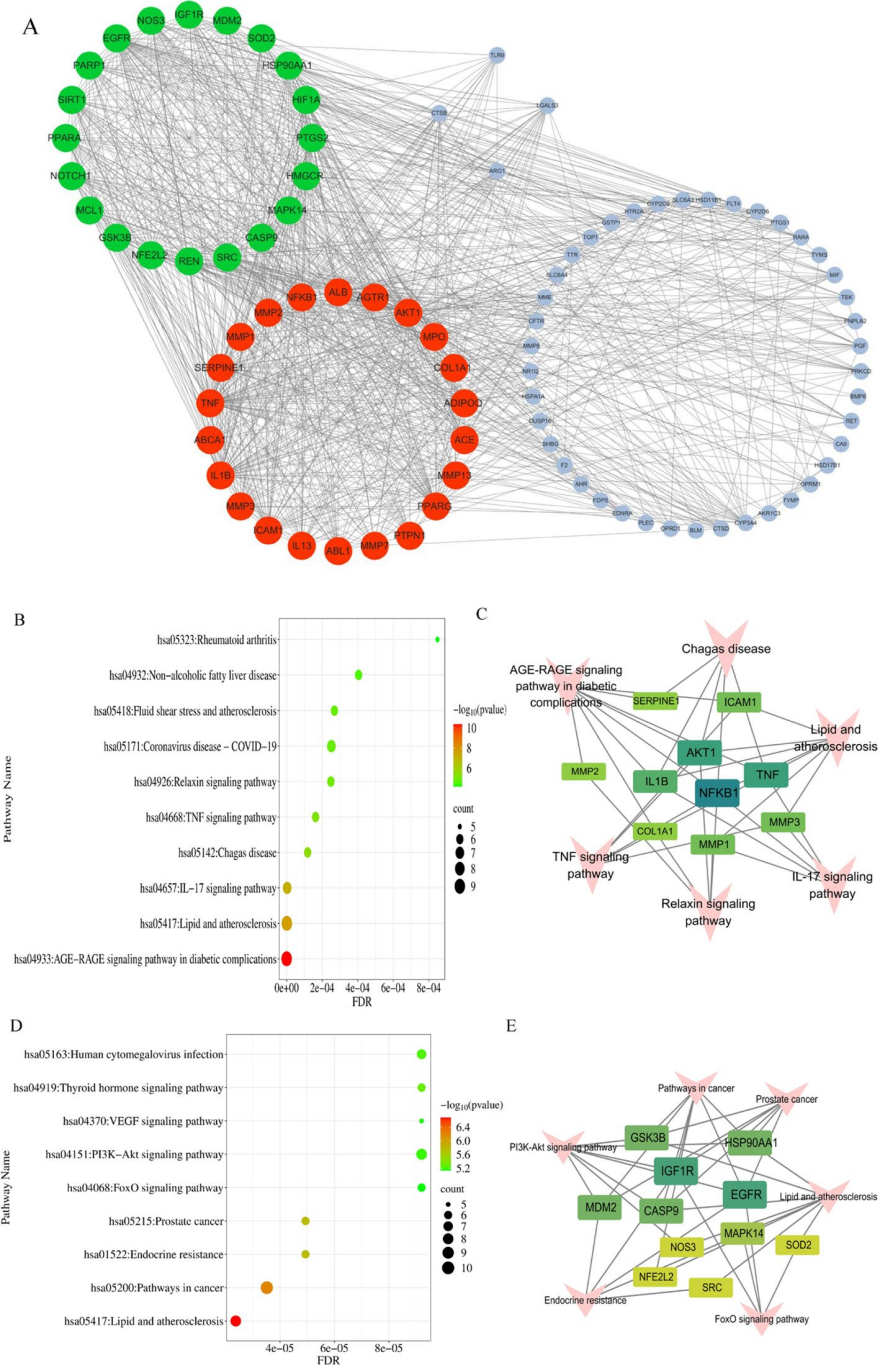
**A **Function modules-based network analysis. cluster1 in red, and cluster2 in green. **B** KEGG enrichment analysis of cluster1 (the top 10 enriched pathway). **C **Target-KEGG pathway network(cluster1). The pink V-shaped nodes represent the pathways, and the green square-shaped nodes represent the targets. **D** KEGG enrichment analysis of cluster2 (the top 10 enriched pathway). **E** Target-KEGG pathway network(cluster2). The pink V-shaped nodes represent the pathways, and the green square-shaped nodes represent the targets

To fully understand the biological function of common targets of IAA mitigation DOR, we analyzed the two associated gene clusters (Cluster 1 and Cluster 2) using the “MCODE” module in Cytoscape 3.10.1 (Fig. 4A). The KEGG-enriched pathways of the two clusters were further analyzed. It was found that the enriched pathways of cluster 1 (Fig. 4B) mainly included the AGE-RAGE signaling pathway in diabetic complications、IL-17 signaling pathway、TNF signaling pathway、Relaxin signaling pathway. The enriched pathways in module 2 (Fig. 4D) were mainly Endocrine resistance、Thyroid hormone signaling pathway、PI3K-Akt signaling pathway、VEGF signaling pathway、FoxO signaling pathway (Supplementary file 4). The critical gene targets involved in the various pathways of gene cluster 1 were NK-κB1, AKT1, TNF, and IL-1β (Fig. 4C), whereas gene family 2 focused on related genes such as FGFR, HSP90AA1, IGF1R, MAPK14, and SOD2 **(**Fig. 4E).

### Disease-pathway-target-drug network map

**Fig. 5:**
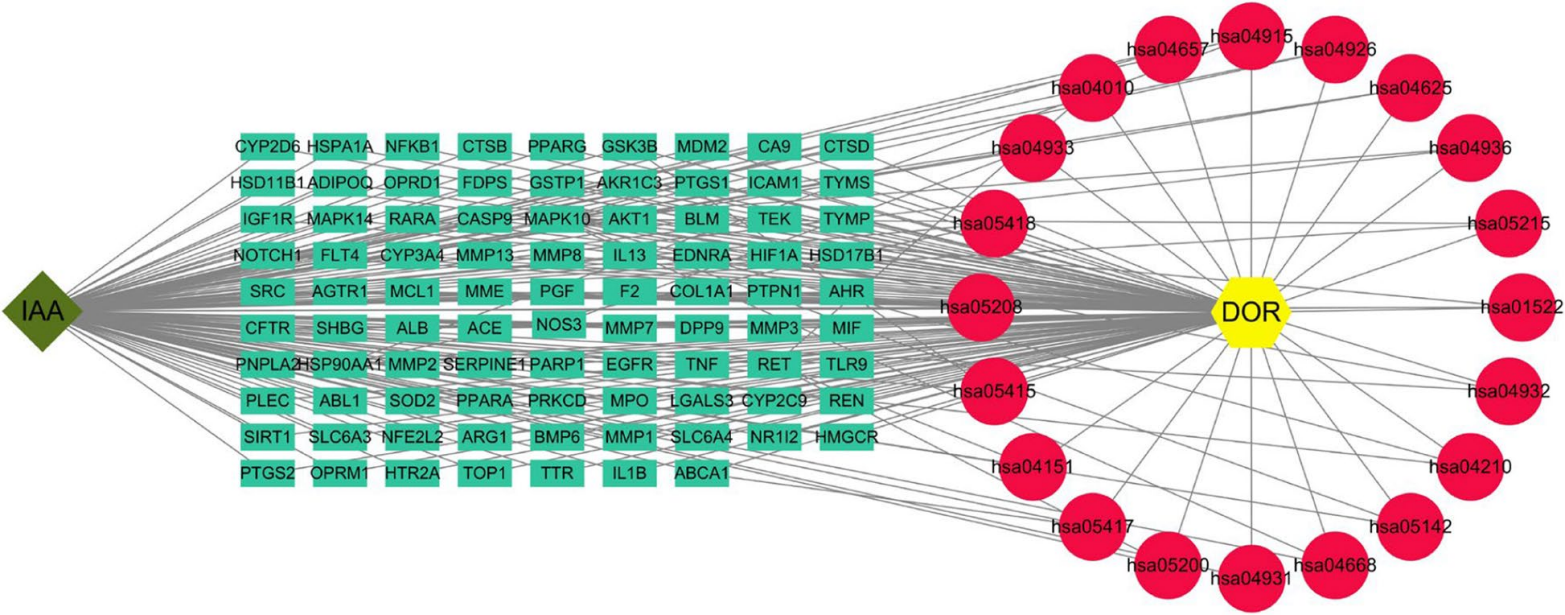
IAA-target genes-pathway-DOR network

Cytoscape 3.10.1 was used to construct a DOR-target gene-pathway-IAA network graph, which consisted of 110 nodes (where yellow nodes represent diminished ovarian reserve, red nodes represent pathways, green nodes represent mutually shared intersecting gene targets, and dark green represents Indole-3-acetic acid) and 235 interaction links (Fig. 5). These targets are distributed in different pathways, and their interactions together determine the mechanism of IAA treatment of DOR. The core pathway-specific information is shown in Table 2.

**Table 2:** The KEGG results

**Pathway number**	**Pathway**	**Count**	***p*-value**
hsa05417	Lipid and atherosclerosis	20	4.5349E-14
hsa05200	Pathways in cancer	26	4.43861E-12
hsa04933	AGE-RAGE signaling pathway in diabetic complications	13	9.21905E-11
hsa04657	IL-17 signaling pathway	12	7.83043E-10
hsa05415	Diabetic cardiomyopathy	15	3.82524E-09
hsa05418	Fluid shear stress and atherosclerosis	13	4.39125E-09
hsa05208	Chemical carcinogenesis - reactive oxygen species	14	1.08079E-07
hsa05215	Prostate cancer	10	2.32979E-07
hsa01522	Endocrine resistance	10	2.54636E-07
hsa04926	Relaxin signaling pathway	11	2.62398E-07
hsa04625	C-type lectin receptor signaling pathway	10	4.2515E-07
hsa04915	Estrogen signaling pathway	11	4.61036E-07
hsa05142	Chagas disease	9	4.09918E-06
hsa04151	PI3K-Akt signaling pathway	15	4.38456E-06
hsa04936	Alcoholic liver disease	10	5.82325E-06
hsa04931	Insulin resistance	9	6.29293E-06
hsa04668	TNF signaling pathway	9	9.4085E-06
hsa04932	Nonalcoholic fatty liver disease	10	1.18876E-05
hsa04010	MAPK signaling pathway	13	1.84147E-05
hsa04210	Apoptosis	9	3.41578E-05

### Molecular docking results

**Fig. 6:**
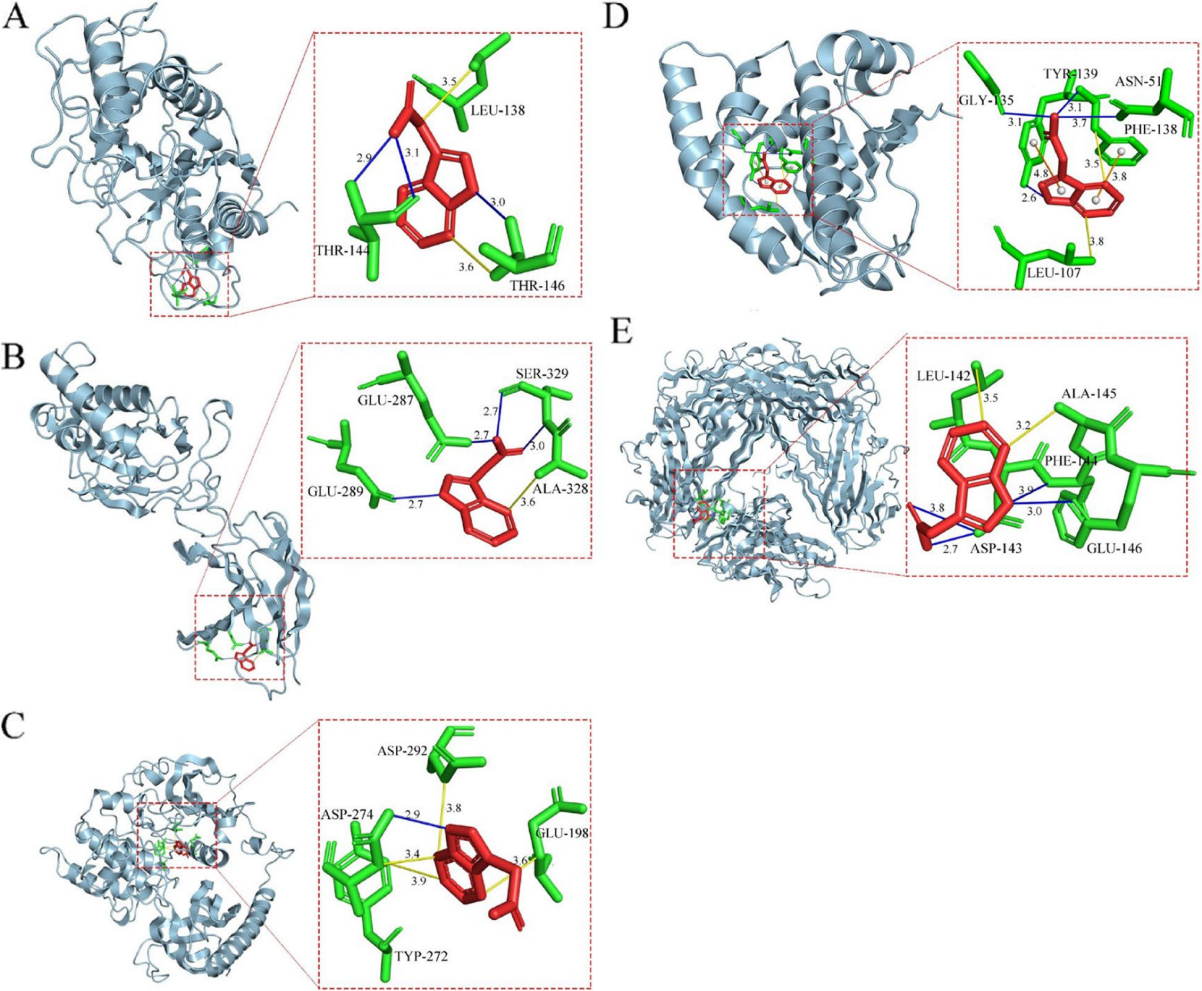
Molecular docking of the top five hub targets with IAA. **A** The binding poses of MMP2 complexed with IAA. **B** The binding poses of NF-κB complexed with IAA. **C** The binding poses of AKT1 complexed with IAA. **D** The binding poses of HSP90AA1 complexed with IAA. **E** The binding poses of TNF-α complexed with IAA. (Blue Line—Hydrogen Bonding, Yellow line-hydrophobic interactions, Orange line—π-stacking)

To explore the possible role of IAA in target proteins associated with diminished ovarian reserve, we used a molecular docking approach to investigate the binding affinity of the top 16 potential core gene targets in the PPI network to IAA. It is generally accepted that the lower the binding energy, the more stable the receptor and ligand binding conformation. A less than −5 kCal/mol binding energy indicates a strong affinity between the two. The following five target proteins were predicted to be most likely to bind to IAA according to the binding affinity from high to low (Table 3), including AKT1(4EJN), MMP2(8H78), HSP90AA1(4BQG), TNF-α(7TA6), and NF-κB(1SVC). This suggests the possibility that IAA affects DOR by regulating the activity of these proteins. Molecular docking visualization results are shown in Fig. 6(A-E). For example, IAA forms hydrogen bonds with MMP2 at amino acid residues THR-144 and THR-146 and has hydrophobic interactions with THR-146 and LEU-138.

**Table 3:** The molecular docking parameters and results

**Serial number**	**Targets**	**PDB ID**	**Box_center(x,y,z)/Å**	**Affinity(kcal/mol)**
1	AKT1	4EJN	-1.54,6.34,-12.65	-7.14
2	MMP2	8H78	12.70,18.66,7.33	-6.47
3	TNF-α	7TA6	0.25,14.84,20,45	-5.99
4	HSP90AA1	4BQG	1.59,16.21,20.45	-5.42
5	NF-κB	1SVC	0.257,14.84,20.45	-5.28

### Molecular dynamic simulation analyses

**Fig. 7:**
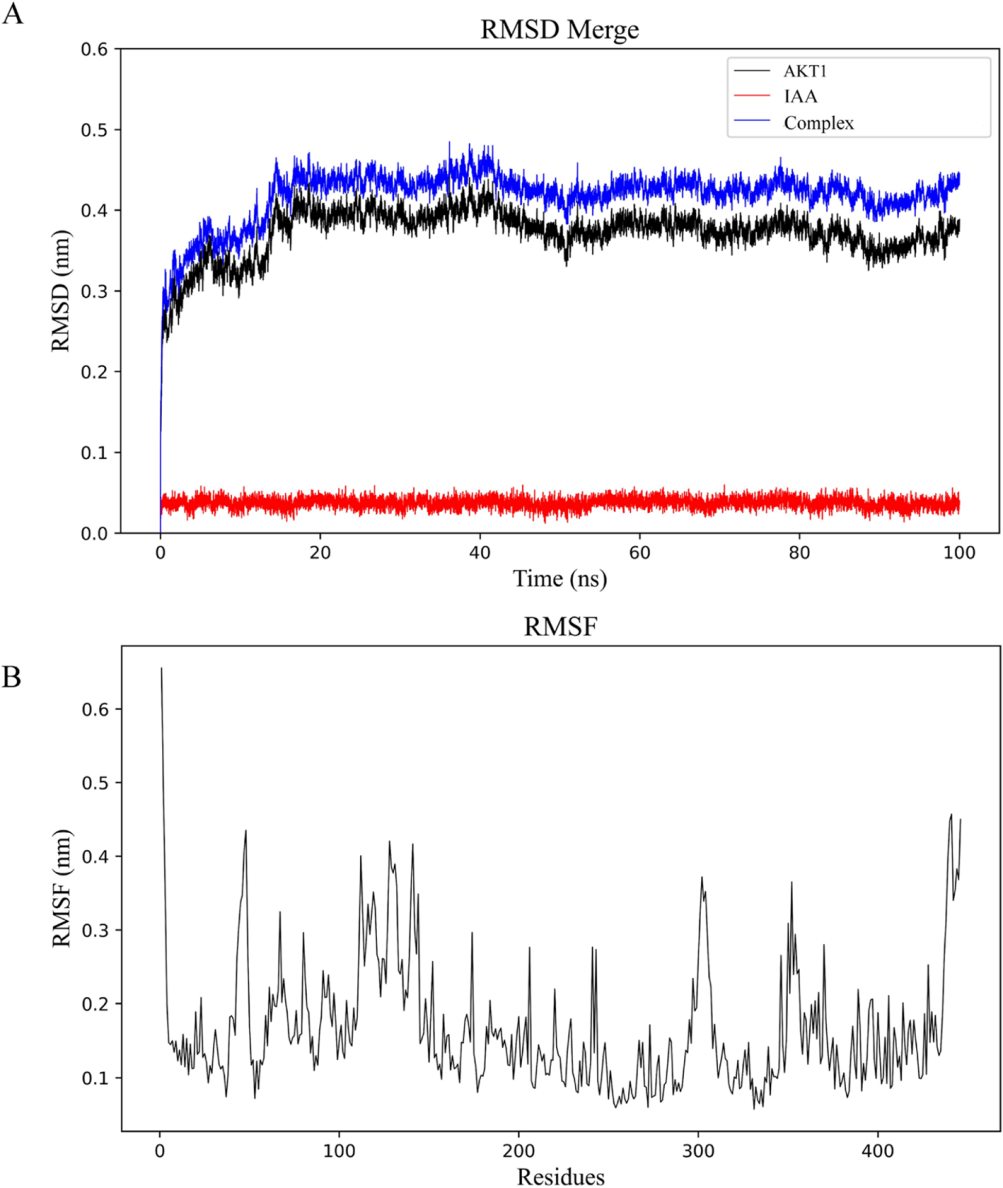
Molecular dynamics simulation between IAA and AKT1 protein. **A** RMSD.**B** RMSF

RMSD is an index to assess the structural changes of proteins. As shown in Fig. 7A, the RMSD of the small molecule protein complex fluctuated smoothly and maintained in a small range after 20 ns, indicating that the binding of AKE1 to IAA was relatively stable. RMSF is an indicator for assessing the dynamics of proteins. The results showed that the RMSF values of amino acid residues fluctuated less in the regions of 190–290 and 390–410 ps (Fig. 7B). In addition, the results of MM/GBSA showed that the free energy of binding of IAA to AKT1 protein was −28.5 ± 0.57 kcal/mol (Table 4).

**Table 4:** Binding free energy calucations by MM/GBSA(kcal/mol)

**Energy**	**AKT1-indole-3-acetic acid**
VDWAALS	-32.73±0.05
ΔE_EL_	-26.65±0.50
ΔE_GB_	-34.71±0.27
ΔE_SURF_	-3.38±0.05
ΔG_gas_	-59.38±0.50
ΔG_solv_	-31.33±0.27
ΔG_MMGBSA_	-28.05±0.57

*VDWAALS *Van der Waals energy, *ΔEEL* Electrostatic energy, *ΔEGB* Polar solvation energy, *ΔESURF* Non polar solvation energy, *ΔGgas* Molecular mechanics term energy, *ΔGsolv* Solvation energy, *ΔGMMGBSA* Binding free energy

### Effect of IAA on the viability of KGN cells

**Fig. 8:**
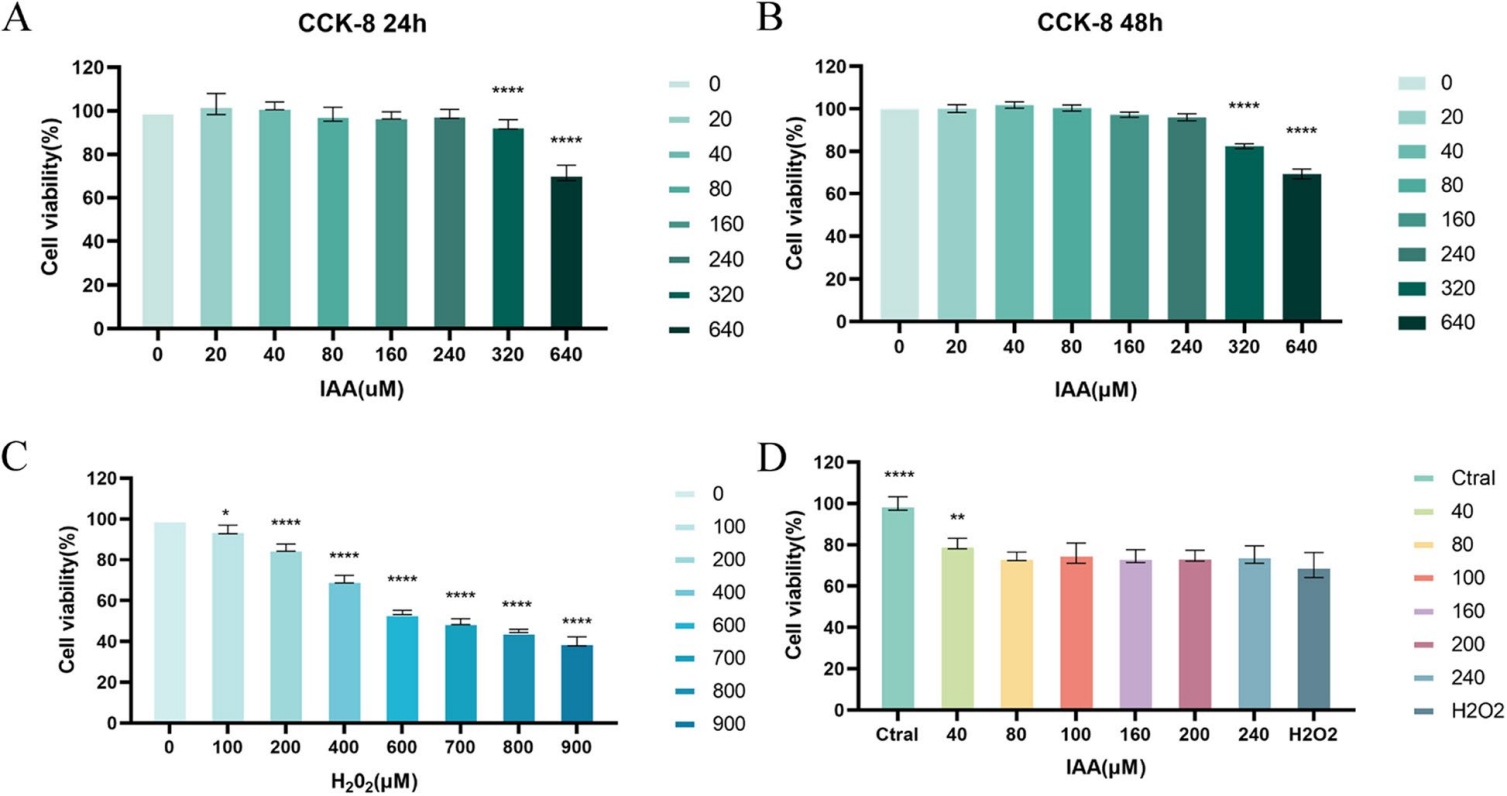
Effect of IAA on the viability of KGN cells. **A**-**B** The viability of KGN cells was assessed using the CCK8 assay after 24 h and 48 h of IAA at the corresponding concentrations. **C** The viability of KGN cells was assessed using CCK8 assay after TBHP treatment. **D** Viability of KGN cells after IAA and TBHP treatment. (*n* = 6), *p < 0.05, **p < 0.01

KGN cells were pretreated with 0, 20, 40, 80, 160, 240, 320, and 640 μ M concentrations of IAA (Aladdin, China, purities:98%, CAS No.: 87–51-4, Item No.: I101074) for 24 h and 48 h, followed by detection of KGN cell viability by CCK8. At concentrations lower than 240 μ M for 24 h and 48 h, IAA had no significant effect on the viability of KGN cells (Fig. 8 A-B). In addition, KGN cells were treated with 0- 900 μ M of tert-Butyl hydroperoxide solution (TBHP (MACKLIN, China, concentration,70% in H2O) for 4 h, as shown in Fig. 8C. Finally, we selected a 600 μ M concentration of TBHP to model oxidative stress. We used IAA to pretreat KGN followed by TBHP action, and cell viability was increased in the 40 μ M IAA pretreated group compared to the model group (*p* < 0.01) (Fig. 8D).

### IAA intervention affects the expression level of each target

**Fig. 9:**
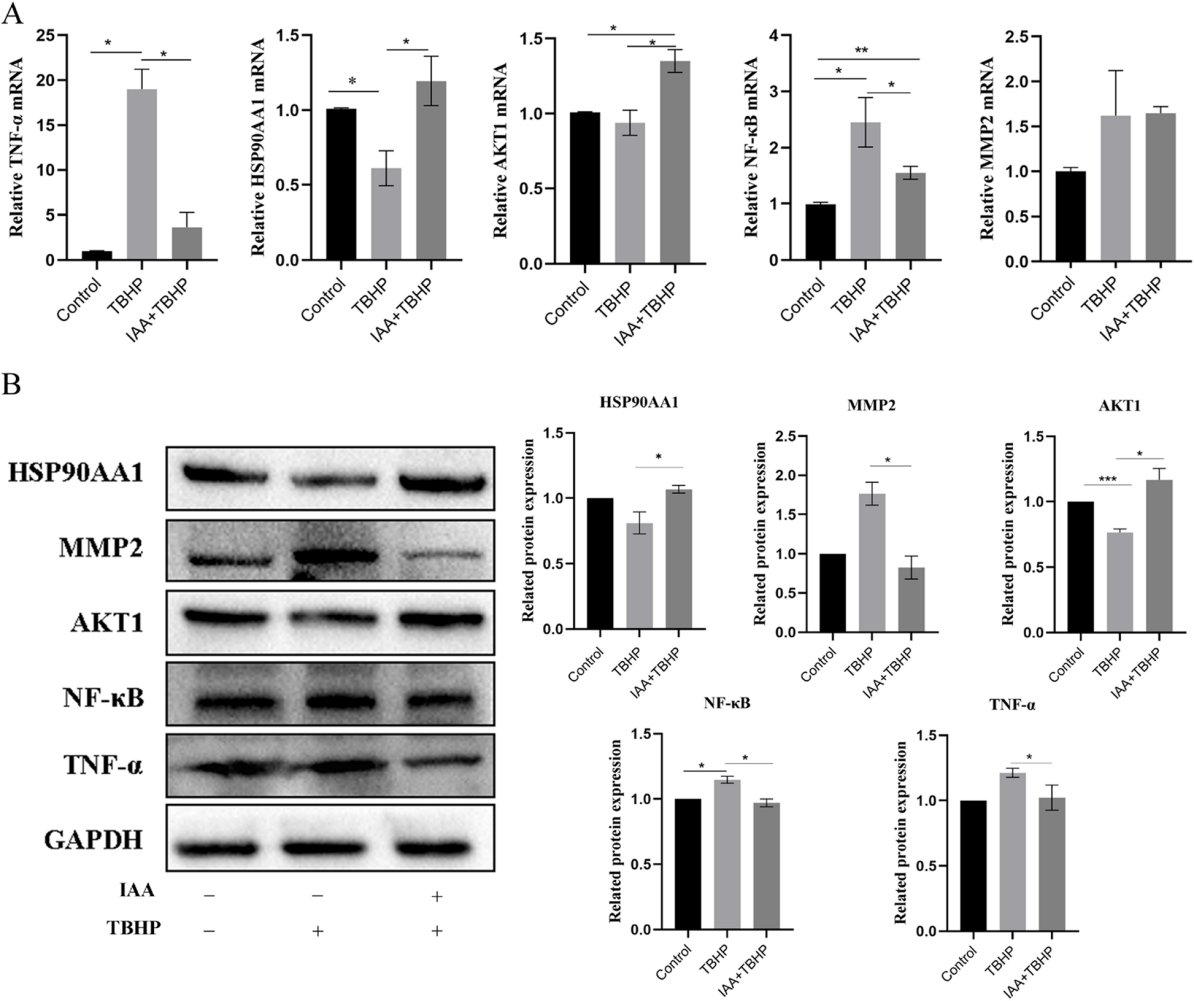
IAA intervention affects the expression level of each target. **A** mRNA expression level of TNF-α, HSP90AA1, AKT1, NF-κB, MMP2 in KGN after IAA intervention. **B** The protein expression level of HSP90AA1, MMP2, AKT1, NF-κB, and TNF-α in KGN after IAA intervention. Data are expressed as mean ± scale (*n* = 3): *p < 0.05, **p < 0.01

The gene and protein expression levels of HSP90AA1, MMP2, TNF, AKT1, and NFκ-B were examined in KGN after treatment with IAA and TBHP. RT-qPCR results showed that compared with the model group, the expression levels of HSP90AA1 and AKT1 were increased in KGN after the administration of IAA (*p* < 0.05), whereas the expression levels of TNF, and NFκ-B significantly decreased (*p* < 0.05) (Fig. 9A). Western blot analysis showed that the protein expression levels of these molecules were also altered (Fig. 9B).

## Discussion

Diminished ovarian reserve (DOR) has a complex etiology and unknown pathogenesis, and its incidence has been on the rise in recent years. Currently, there is a lack of effective methods to treat DOR, so seeking effective treatments for DOR has become one of the current research hotspots in the reproductive neighborhood [7]. Tryptophan is an essential amino acid in humans, obtained only through diet, and is widely involved in various physiological processes such as protein synthesis, inflammatory response, oxidative stress, and intestinal homeostasis. Tryptophan has three main metabolic pathways in the human body: the kynurenine pathway, the 5-hydroxytryptamine pathway, and the indole pathway, of which intestinal microorganisms metabolize the indole pathway. IAA is an indole-ring containing metabolite produced by intestinal microorganisms to metabolize tryptophan, and it has anti-inflammatory and antioxidant properties. In rats, *L. rhamnosus* may exert anti-inflammatory effects by up-regulating tryptophan metabolism and increasing the IAA content to treat acne vulgaris [31]. In cellular experiments, IAA attenuated LPS-induced inflammatory responses and free radical production in RAW264.7 macrophages by inducing heme oxygenase-1 (HO-1) and directly neutralizing free radicals [32].

Interestingly, our previous study found that IAA levels in follicular fluid (FF) were significantly lower in patients with DOR than in controls and suggested that IAA may be a potential biological marker or overprotective agent for DOR [14]. Overall, it is reasonable to assume that IAA may play a potential role in improving ovarian function. However, the biological pathways and specific mechanisms of its action are still unclear. In this study, for the first time, the mechanism of action of IAA on DOR was revealed using systematic network pharmacology and bioinformatics, 16 hub target genes were obtained, and further molecular docking was performed. According to network pharmacology and molecular docking results, TNF-α, NF-κ B, AKT1, MMP2, and HSP90AA1 play crucial roles in improving DOR by IAA.

Tumor necrosis factor (TNF-α) is an essential factor that participates in and maintains the inflammatory response in vivo, and TNF-α induces the expression of inflammatory genes to directly drive the inflammatory response or indirectly by causing cell death [33]. TNF-α is widely expressed in the reproductive system, including ovarian granulosa and endometrial cells. Its expression is subject to highly integrated endocrine, paracrine, and autocrine mechanisms. Its expression is regulated by highly integrated endocrine, paracrine, and autocrine mechanisms. It is crucial in maintaining granulosa cell survival, regular ovulation, follicular development, and atresia [34, 35]. However, imbalanced TNF-α expression is closely associated with polycystic ovary syndrome, diminished ovarian reserve function, ovarian senescence, and granulosa cell apoptosis [36–41]. A previous study showed that the expression of TNF-α and related pro-inflammatory factors was significantly increased in the follicular fluid of patients with diminished ovarian reserve (DOR) compared to those with usual ovarian reserve (NOR) [39]. Existing studies have shown that intervention treatment with IAA significantly reduces the expression level of TNF-α in NAFLD, dental pulp stem cells, and rat model of acne vulgaris [31], thereby attenuating the inflammatory response and oxidative stress levels [11, 42]. In the present study, IAA advance intervention significantly reduced the expression level of TNF-α in oxidative stress-impaired KGN cell*s.*

NF-κB can be activated by various pathological factors and is involved in regulating the expression of inflammatory factors and various enzymes involved in amplifying the inflammatory response cascade (e.g., COX-2, iNOS, etc.) [43]. Abnormal activation of the NF-κB signaling pathway in vitro experiments involves oxidative stress, apoptosis, and other biological processes in granulosa cells, closely related to decreased ovarian function and premature ovarian aging [44, 45]. The intervention of IAA could reverse the expression of NF-κB in the modeled KGN cells, and it was hypothesized that IAA might attenuate the ovarian inflammatory response by down-regulating the NF-κB signaling pathway, thereby protecting the ovarian function.

AKT1 is a serine/threonine protein kinase widely expressed in human primordial follicles, follicles at all stages of growth, and granulosa cells. It involves biological processes such as primordial activation, secondary follicle development, and oocyte survival [46, 47]. It has been reported that AKT1 plays an essential role in regulating ovarian growth and maturation and that AKT1 deficiency leads to premature ovarian failure in female mice [48]. Abnormal PI3K/AKT signaling pathway is closely related to pathological changes in the ovary. Reduced activity of the PI3K/AKT pathway promotes the translocation of Bax to the mitochondria, followed by the release of cytochrome C, which triggers apoptosis through the caspase pathway [49], resulting in impairment of ovarian function. Early intervention of IAA alleviated the H2O2-induced elevation of AKT expression levels to a certain extent or may play a positive role in mitigating DOR by influencing the expression of other molecules through AKT1. Meanwhile, the molecular docking results showed that hydrogen bonds were formed between ASP274 amino acid residues on AKT1 and hydrophobic forces were generated by residues GLU-198, TYR-272, ASP274, and ASP292, which are located in the catalytic structural domains of AKT1, and may have a specific effect on the activity of the AKT1 kinase; therefore, we hypothesized that IAA could improve the ovarian function by regulating AKT levels to improve ovarian function, but further experimental verification is needed.

MMP2 is a ubiquitin metalloproteinase involved in various biological functions in the human body, such as vascular system remodeling, angiogenesis, tissue repair, tumor infiltration, inflammation, and atherosclerotic plaque rupture. Matrix metalloproteinases (MMP) play an essential role in the dynamic process of folliculogenesis [50], while hormone levels regulate the type of MMP expression. Studies have shown that MMP2 is the most abundant MMP in cat follicles [51], and secretion of MMP2 and MMP9 by granulosa cells to catabolize type VI collagen components of the extracellular matrix plays a vital role in tissue remodeling during follicular growth and development especially in the formation and expansion of sinusoidal lumen in the ovarian mound complex and ovulation [51, 52]. A study showed that MMP2 is more expressed in the ovarian mound and granulosa cells in the presence of reduced ovarian response and decreased fertilization [53]. Similarly, another study showed elevated levels of MMP2 expression in senescent porcine granulosa cells [54]. This suggests that MMP2 may be a potential target for ovarian dysfunction, and in this study, IAA early intervention alleviated some of the H2O2-induced elevated expression levels of MMP2.

Heat shock protein 90 (Hsp90) is a well-characterized molecular chaperone protein. Unlike the constitutive expression of Hsp90β, Hsp90α (HSP90AA1) is the stress-induced isoform [55], which binds to phospholipids to stabilize the cell membrane structure [56], resists cellular stress and helps normal cells to fold proteins into the correct spatial structure during heat stress thereby executing the appropriate biological functions. Hsp90AA1 can maintain cell survival during heat stress by inhibiting apoptosis and increasing cell autophagy [57]. A study found that a decrease in Hsp90AA1 decreases the rate of in vitro maturation, cleavage, and blastocyst formation in bovine oocytes, as well as increasing cleavage rates and affecting embryo quality [58]. Hsp90 plays a vital role in sperm development, and males of certain trans-heterozygous combinations with mutant Hsp90AA1 alleles have been described to exhibit sterility and show disruption of meiosis in Drosophila [59]. In mice, the Hsp90 protein assists tNASP (testicular histone-binding protein) folding into the correct spatial structure and performing the appropriate function [60–62]. In the present study, IAA early intervention alleviated the H2O2-induced decrease in the expression level of HSP90AA1 compared to the model group.

In conclusion, there is no direct evidence from animal and clinical studies that IAA can improve DOR through the above five targets; however, the molecular docking results of the present study confirmed that IAA has good binding ability to all five of the six targets mentioned above, and molecular dynamics simulations further proved the stability of the binding between AKT1 and IAA. Combined with the results of the in vitro experiments and its physiological functions in the ovary, we hypothesized that IAA could benefit the ovary by affecting these targets and pathways. These results can provide a preliminary reference and basis for future primary research.

We used a network pharmacology approach to investigate the molecular mechanisms by which IAA affects DOR. Eighty-eight common targets and 16 core target proteins were analyzed by GO enrichment, and these genes were mainly involved in biological processes such as inflammatory response, regulation of cell proliferation and apoptosis, hypoxia response, regulation of enzyme activities, and signal transduction. In addition, KEGG pathway analysis showed that the AGE-RAGE signaling pathway in diabetic complications, IL-17 signaling pathway, 、Chemical carcinogenesis—reactive oxygen species were the three most enriched signaling pathways.

The interaction of advanced glycosylation end products (AGEs) and their receptor RAGE can activate various signaling pathways, such as NF-κB, MAPK, and PI3K-AKT-mTOR, leading to inflammatory responses, oxidative stress, and other adverse effects, which are closely related to ovarian aging [63]. Qiao-Li Zhang et al. found that, compared with mice on a regular diet, female mice on a high-sugar diet showed disturbed estrous cycles, significantly reduced numbers of antral and sinus follicles, and inhibited primary follicular growth [64]. Similarly, human IVM oocytes on a high-sugar diet showed a significantly lower rate of first-polar body extrusion and abnormal levels of DNA methylation [65]

Inflammatory responses play a critical role in physiologic events such as the female menstrual cycle, embryo implantation, pregnancy, and childbirth and are intricately related to folliculogenesis and ovulation. Dysregulation of these regulatory processes can lead to a decline in oocyte quality and affect fertility [66]. Ovarian aging is closely related to inflammation, and Carolina Lliberos et al. [67] found that serum concentrations of several pro-inflammatory cytokines (TNF-α, IL-6, inflammatory vesicle genes ASC and NLRP3) and mRNA levels in the ovary increased significantly with age in mice. Also, the levels of pro-inflammatory factors such as IL-1β, IL-6, and IL-21 were increased, and the level of anti-inflammatory factor IL-4 was decreased in the follicular fluid of POI patients compared to healthy controls [68]. IL-17 has been reported to stimulate the release of inflammatory factors such as IL-6, TNF-α, IL-β, IFN-γ, etc., through multiple cascade effects of the MAPK pathway (including p38, ERK, and INK pathways) and the NF-κB signaling pathway. In addition, it was found that IL-17A/IL-6 axis expression was up-regulated in a cyclophosphamide (CTX)-induced model of premature ovarian failure in rats, significantly regulating the activities of REK1/2 and MEK1/2 [69].

In addition to the above 2 pathways, we also paid particular attention to the role of Chemical carcinogenesis—reactive oxygen species signaling in IAA anti-DOR. Normal levels of ROS in ovarian tissues play an essential regulatory role in normal follicular development, steroid hormone synthesis, and angiogenesis. However, excessive accumulation of ROS causes DNA damage in granulosa cells, reduction of base oxidation and gene repair, mitochondrial dysfunction, abnormal meiosis in oocytes, and shortening of telomerase, which leads to apoptosis of GC cells, follicular atresia, and reduction of oocyte quality, which in turn leads to ovarian aging and diminished ovarian reserve function. This leads to GC cell apoptosis, follicular atresia, and oocyte quality reduction, leading to ovarian senescence and diminished ovarian reserve function [70]. Meanwhile, high levels of ROS can mediate multiple signaling pathways, such as phosphatidylinositol 3-kinase/protein kinase B (PI3K/Akt), mitogen-activated protein kinase (MAPK), and Kelch-like ECH-associated protein 1 (Keap1)-like protein, nuclear factor erythroid 2-related factor 2 (Nrf2)-antioxidant response elements (ARE), NF-κB signaling pathway, and FOXO axis, which are involved in biological processes such as ovarian oxidative stress injury, granulosa cell apoptosis, follicular atresia, and diminished ovarian reserve function [43]. Excessive ROS accumulation leads to ovarian dysfunction; thus, it is crucial to maintain a dynamic balance between ROS and antioxidant systems by reducing ROS production and increasing the activity of endogenous antioxidant systems on the one hand, and supplementing with exogenous antioxidants to enhance the ability to scavenge excess oxygen radicals produced on the other. Previous studies have shown that IAA has been found to have anti-inflammatory and antioxidant effects in tissues such as the liver and kidney, and combined with the above analyses, we initially hypothesized that IAA may be able to treat DOR by exerting anti-inflammatory and antioxidant effects.

## Conclusions

In conclusion, through network pharmacology and molecular docking, we have elucidated the possible molecular mechanisms of IAA to treat diminished ovarian reserve and identified some of its target actions that may benefit DOR. However, these data support the idea that additional in vivo and in vitro experiments are still needed to further elucidate its function and mechanism of action.

## Supplementary Information

Supplementary Material 1.

Supplementary Material 2.

Supplementary Material 3.

Supplementary Material 4.

Supplementary Material 5.

## Data Availability

No datasets were generated or analysed during the current study.
