# Supplementary material for: Predicting the therapeutic role and potential mechanisms of Indole-3-acetic acid in diminished ovarian reserve based on network pharmacology and molecular docking
Source: Hereditas. 2024 Nov 21;161:47. doi: 10.1186/s41065-024-00348-6 (PMC11580193; doi:10.1186/s41065-024-00348-6)
Supplement: Supplementary file 1 — Supplementary Material 1. [file 41065_2024_348_MOESM1_ESM.pdf]

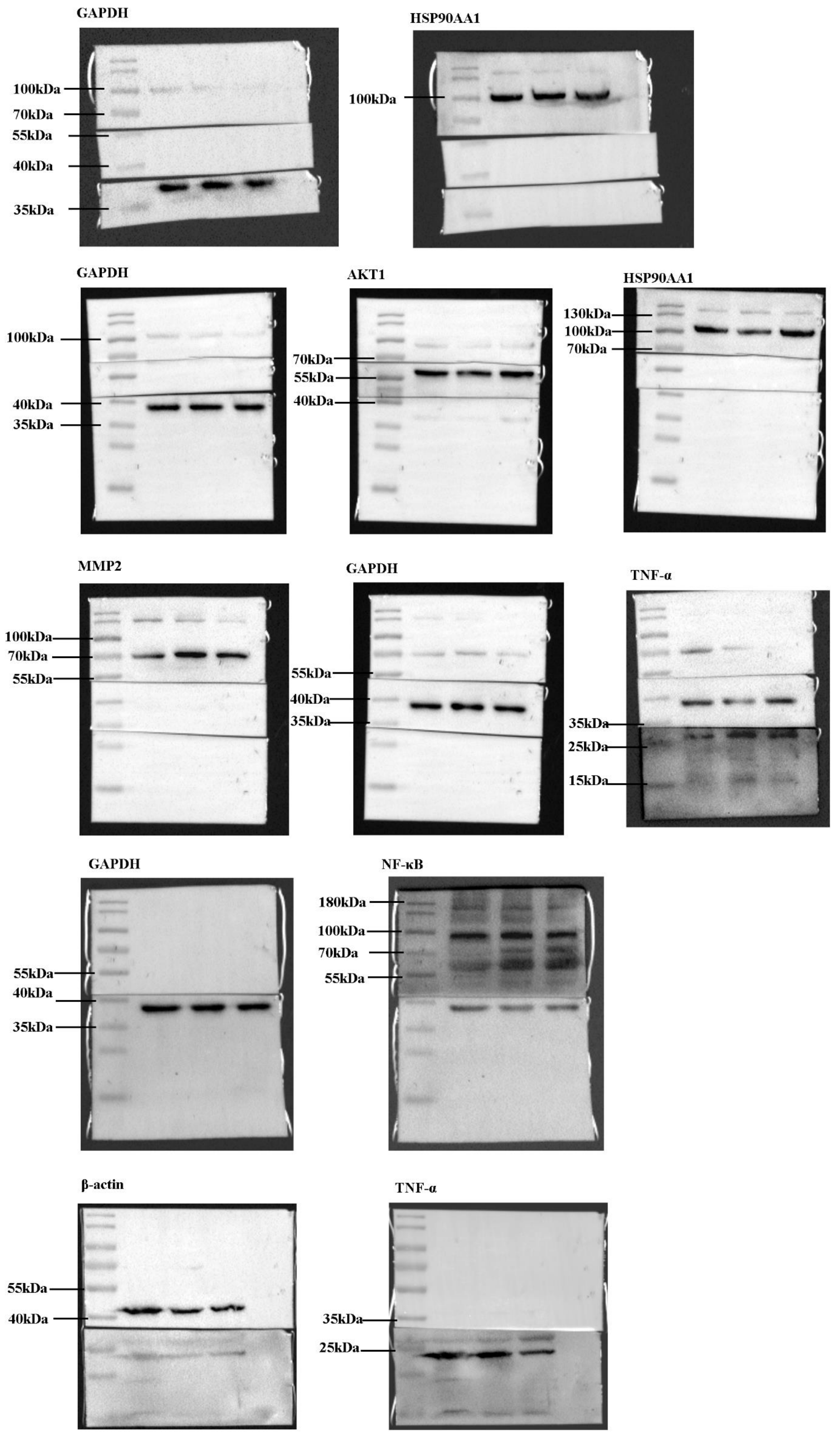

GAPDH
100kDa
70kDa
55kDa
40kDa
35kDa
HSP90AA1
100kDa
GAPDH
100kDa
40kDa
35kDa
AKT1
70kDa
55kDa
40kDa
HSP90AA1
130kDa
100kDa
70kDa
MMP2
100kDa
70kDa
55kDa
GAPDH
55kDa
40kDa
35kDa
TNF-α
35kDa
25kDa
15kDa
GAPDH
55kDa
40kDa
35kDa
NF-κB
180kDa
100kDa
70kDa
55kDa
β-actin
55kDa
40kDa
TNF-α
35kDa
25kDa

HSP90AA1

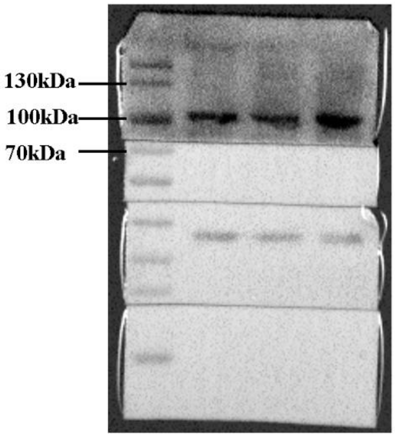

AKT1

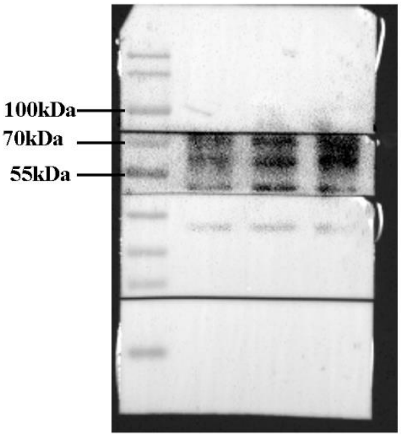

GAPDH

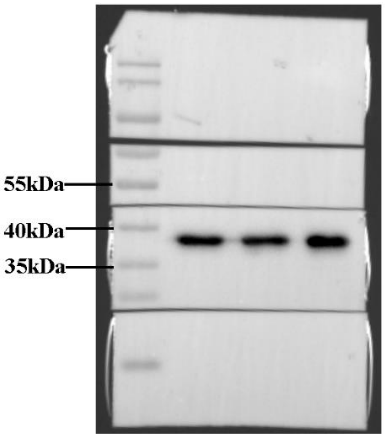

GAPDH

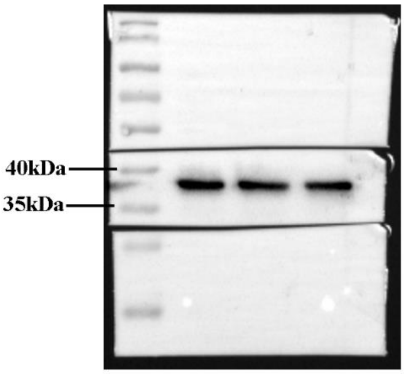

MMP2

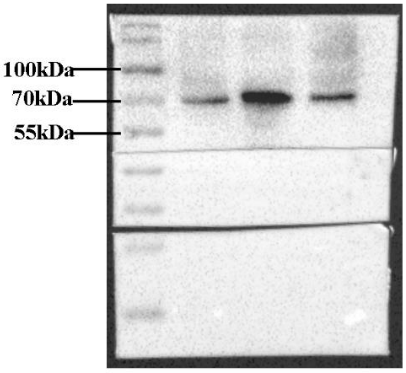

GAPDH

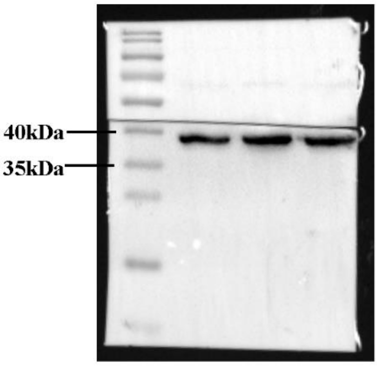

AKT1

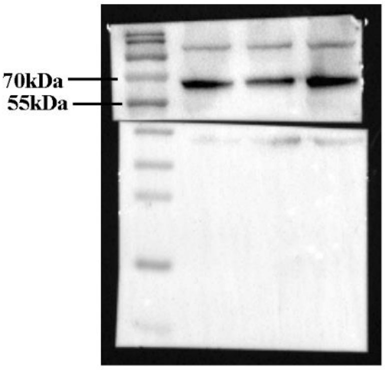

GAPDH

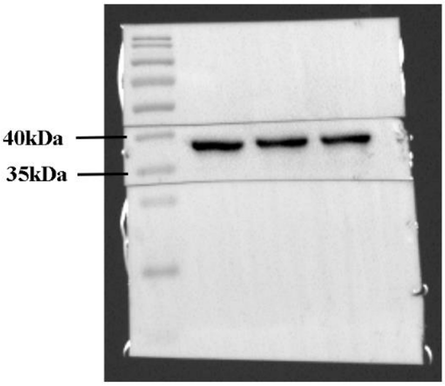

MMP2

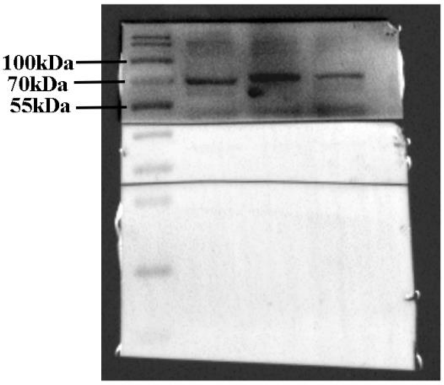

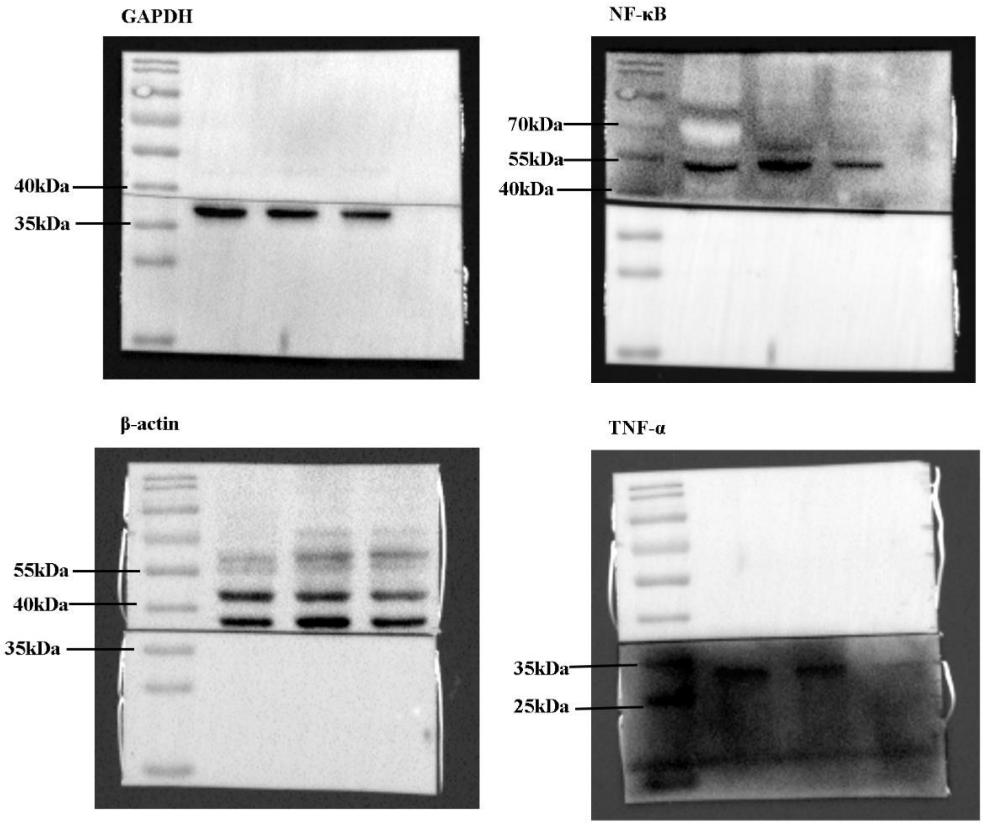
GAPDH
40kDa
35kDa
NF-κB
70kDa
55kDa
40kDa
β-actin
55kDa
40kDa
35kDa
TNF-α
35kDa
25kDa

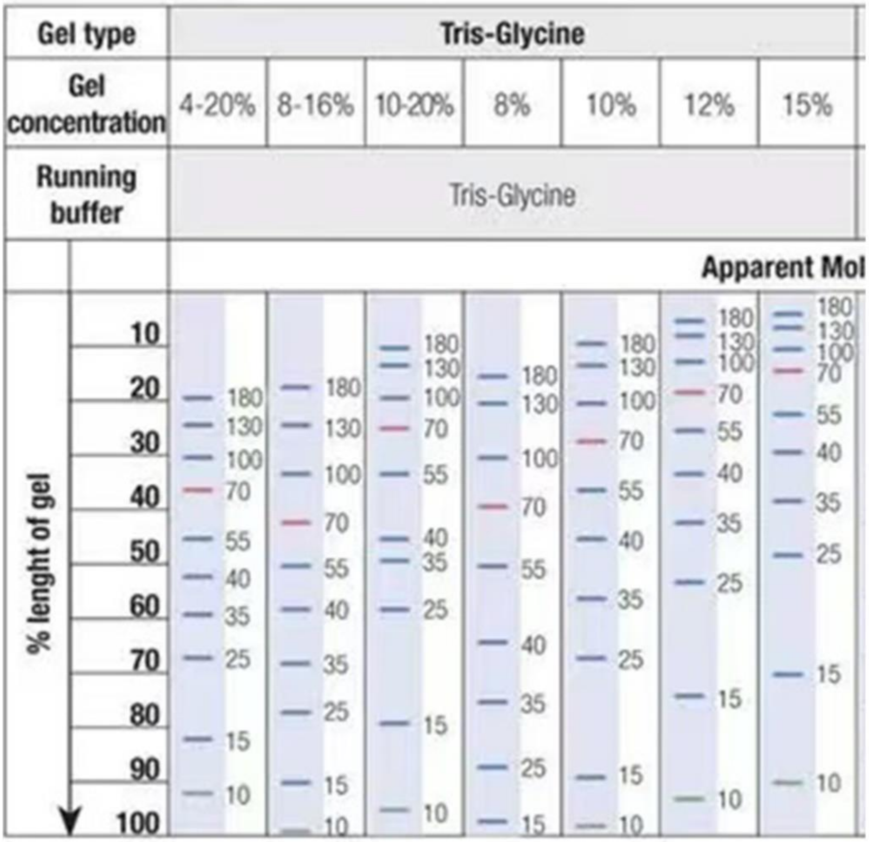
Gel type
Tris-Glycine
Gel concentration
4-20%
8-16%
10-20%
8%
10%
12%
15%
Running buffer
Tris-Glycine
Apparent Mol
% lenght of gel
10
20
30
40
50
60
70
80
90
100
180
130
100
70
55
40
35
25
15
10
